# Reply: Repair or replace the aortic root: The eternal unsolved dilemma

**DOI:** 10.1016/j.xjon.2022.04.030

**Published:** 2022-04-23

**Authors:** Grace Lee, Veronica Chan, Bobby Yanagawa

**Affiliations:** Division of Cardiac Surgery, St Michael's Hospital, University of Toronto, Toronto, Ontario, Canada

Reply to the Editor:

**Figure:**
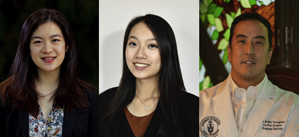

The authors reported no conflicts of interest.

The *Journal* policy requires editors and reviewers to disclose conflicts of interest and to decline handling or reviewing manuscripts for which they may have a conflict of interest. The editors and reviewers of this article have no conflicts of interest.

We have a greater appreciation of the aortic root as a complex living, functional unit comprising interdependent elements, and this knowledge is guiding our surgical approach. Pradegan and colleagues^1^ touched on some important points in regards to selective aortic sinus replacement^2^ and highlighted some important and unresolved questions.

First, is there any advantage to single or double sinus replacement versus complete root replacement? Yes, we agree with the authors. Isolated sinus replacement, especially nonsinus replacement, is faster, simpler, and associated with no risk of technical or bleeding concerns with coronary buttons. There is less overall prosthetic material with at least a theoretical lower risk of infective endocarditis. Thus, if the long-term outcome is comparable vis-à-vis aortic valve function and stability of root size, then limited sinus replacement would be a very reasonable and perhaps even a preferred approach.

Second, those patients with cusp prolapse/pseudo cusp prolapse were at risk of moderate-to-severe aortic insufficiency in follow-up. Previous studies have shown leaflet elongation to be an initially adaptive response to aortic root dilation.^3^ Our understanding of long-term outcomes of aortic cusp repair strategies lags that of the mitral valve. Greater understanding of the optimal measure of cusp geometry—whether that is intercommissural distance, length of cusp insertion, amount of cusp tissue (eg, geometric height), or length of free margin—as well as optimal repair technique will be important here.

Third, the authors propose an interesting hypothesis as to why the noncoronary cusp is more prone to dilation than the left or right coronary cusps. Typically, the right sinus is largest and tallest, followed by the non- then left coronary sinus.^4^ In fact, the sinuotubular junction is not quite parallel to the aortic annulus and has a slight tilt in the right/anterior aspect. However, the left and right coronary cusps are buttressed by the coronary os, and Pradegan and colleagues^5^ report increased peri-os collagen fibers (seen in the context of bicuspid aortic valve), which may protect the sinus from dilation.

This series of selective sinus replacement by Urbanski and colleagues^2^ offers insights into a potential tool for surgeons. We agree with Pradegan and colleagues that more work is needed to identify the ideal patient population for this innovative, less-is-more approach for the aortic root.
